# Influenza H7N9 Virus Hemagglutinin with T169A Mutation Possesses Enhanced Thermostability and Provides Effective Immune Protection against Lethal H7N9 Virus Challenge in Chickens

**DOI:** 10.3390/vaccines11081318

**Published:** 2023-08-02

**Authors:** Taoran Chen, Dexin Kong, Xiaolong Hu, Yinze Gao, Shaorong Lin, Ming Liao, Huiying Fan

**Affiliations:** 1 College of Veterinary Medicine, South China Agricultural University, Guangzhou 510642, China; 2 Key Laboratory of Zoonosis Prevention and Control of Guangdong Province, Guangzhou 510642, China; 3 Key Laboratory of Animal Vaccine Development, Ministry of Agriculture, Guangzhou 510642, China; 4 National and Regional Joint Engineering Laboratory for Medicament of Zoonosis Prevention and Control, Guangzhou 510642, China

**Keywords:** H7N9 avian influenza virus, mutant HA protein, baculovirus expression system, thermostability, subunit vaccine

## Abstract

H7N9 avian influenza virus (AIV) has caused huge losses in the poultry industry and impacted human public health security, and still poses a potential threat. Currently, immune prevention and control of avian influenza relies on traditional inactivated vaccines; however, they have some limitations and genetically engineered avian influenza subunit vaccines may be potential candidate vaccines. In this study, a T169A mutation in the HA protein derived from H7N9 AIV A/Chicken/Guangdong/16876 (H7N9-16876) was generated using the baculovirus expression system (BVES). The results showed that the mutant (HAm) had significantly increased thermostability compared with the wild-type HA protein (HA-WT). Importantly, immunizing chickens with HAm combined with ISA 71VG elicited higher cross-reactive hemagglutination inhibition (HI) antibody responses and cytokine (IFN-γ and IL-4) secretion. After a lethal challenge with heterologous H7N9 AIV, the vaccine conferred chickens with 100% (10/10) clinical protection and effectively inhibited viral shedding, with 90% (9/10) of the chickens showing no virus shedding. The thermostability of HAm may represent an advantage in practical vaccine manufacture and application. In general, the HAm generated in this study represents a promising subunit vaccine candidate for the prevention and control of H7N9 avian influenza.

## 1. Introduction

In early 2013, the H7N9 AIV crossed species boundaries to infect humans in China [1]. Since then, it has continued evolving and caused five epidemic waves of human infections, with 1568 cases in total and at least 616 deaths [2,3,4]. Due to its dual receptor-binding feature, H7N9 AIV has posed severe challenges to public health safety and the poultry industry [5,6,7]. Fortunately, H7N9 avian influenza in China has been effectively controlled through the large-scale vaccination of chickens implemented nationwide since September 2017 [8,9]. Until now, prevention and control of H7N9 avian influenza in China have relied mainly on traditional inactivated virus vaccines. However, these conventional vaccines have some shortcomings, including the biosafety risk of virus transmission and the time-consuming production process [10,11]. Therefore, developing a next-generation vaccine that can effectively avoid the above drawbacks is of great significance.

A genetically engineered subunit vaccine is one of the promising candidate vaccines; it has unique advantages that include being free of viral nucleic acid and having reduced immune side effects and the capacity to distinguish vaccine-immunized animals from virus-infected animals [12,13,14]. At present, the most used immunogen for AIV subunit vaccines is hemagglutinin (HA), the main trimeric glycoprotein on the surface of the influenza virus. Using different expression systems, many studies have demonstrated that the HA subunit vaccine possesses the potential to be a candidate vaccine [15,16,17,18,19]. However, the successful development of a commercial subunit vaccine remains challenging. To enhance the immunogenicity of HA, scholars have attempted various strategies to modify the immunogen. A research group found that HA derived from wild-type H3N2 had increased thermal stability compared with a mutant HA containing two cysteine mutations in the transmembrane domain (TM) [20,21,22]. Interestingly, when the TM of H3N2 displaced the TMs of other HAs subtypes, including H1, H5, H7, and H9, the recombinant HA showed improved hetero-protection [23,24,25,26,27]. The above studies suggested that a probable correlation exists between antigen stability and immunogenicity. Additionally, current vaccines must be stored and transported under low-temperature environments because most biological materials maintain normal activity in vitro only under low-temperature conditions [28]. Therefore, improving the thermal stability of proteins is also significant for vaccine production and application.

Some previous studies had proved that specific HA amino acid residues affect the properties of the HA protein, which may further affect the vaccine’s protective efficacy. One research reported that particular mutations (L217Q and A151T) in field isolates collected in 2019 dramatically changed the acid and thermal stabilities of H7N9 HA [29]. Another group’s research reported that one H7N9 virus-like particle (VLP) vaccine, which is composed of HA protein with both avian (G228) and human (L226) receptor specificities, showed improved immunogenicity and protective effect in mice compared with the VLP vaccine containing HA with either L226 + S228 or Q226 + S228 [30]. Based on the above studies, we chose several potential mutation sites in the AIV H7N9-16876 and tested the impact of these sites on HA protein. We identified a potential mutation site, A169T, that may reduce the thermal stability of the HA protein and hypothesized that reverse mutation of this site could enhance the thermal stability of the HA protein, thereby improving its immunogenicity.

In this study, a mutant with the T169A substitution in the HA protein of the highly pathogenic avian influenza virus (HPAIV) H7N9-16876 was prepared by overlapping PCR and BVES. Subsequently, the differences in thermostability and immunogenicity of the mutant (HAm) and wild-type HA (HA-WT) proteins were evaluated. Our results demonstrated that HAm possesses significantly enhanced thermostability and immunogenicity. Compared with immunization with the HA-WT, a single-dose immunization with HAm in conjunction with ISA 71VG induced a higher cross-reactive HI antibody response and cytokine (IFN-γ and IL-4) secretion in chickens. Furthermore, HAm conferred complete clinical protection against lethal challenge with a heterologous H7N9 AIV and effectively inhibited virus shedding in birds.

## 2. Materials and Methods

### 2.1. Cell lines, Viruses, and Plasmids

Sf9 insect cells were cultured in Sf-900TM II SFM (Gibco, USA) at 27 °C. The HPAIV A/Chicken/Qingyuan/E664/2017 (H7N9-E664), A/Chicken/Guangdong/E157/2017 (H7N9-E157), and A/Chicken/Guangdong/16876/2016 (H7N9-16876) used in this study belong to the different sub-branches of the Yangtze River Delta lineages. They were conserved and provided by the College of Veterinary Medicine of South China Agricultural University (SCAU). The codon-optimized HA gene derived from HPAIV H7N9-16876 was synthesized by BGI (Shenzhen, China) and cloned into the cloning vector pUC57.

All experiments involving live H7N9 AIV were carried out in biosafety level 3 (P3) facilities at SCAU.

### 2.2. Gene Mutation and Recombinant Baculovirus Construction

Based on the HA gene of H7N9-16876, the mutant HA gene with a base mutation of A to G at site 505 was generated using overlapping PCR, which encoded a mutant HA protein with a threonine to alanine amino acid substitution at residue 169 (T169A). The primer sequences used for overlapping PCR are given in Table 1. Both the mutant HA gene and the unmutated HA gene were further modified to contain a Kozak sequence (GCCACC) at the N-terminus and a six-fold histidine tag at the C-terminus and then cloned into the multiple cloning site (MCS) of the pACEBac1 vector. The recombinant clones were identified by restriction enzyme digestion. Subsequently, positive plasmids were transformed into *Escherichia coli* DH10Bac for gene transposition and the positively transposed bacmids carrying the gene of interest were used to transfect the Sf9 cells to generate the recombinant baculoviruses. The recombinant baculoviruses were purified via plaque isolation and titrated using plaque assays.

### 2.3. Indirect Immunofluorescence Assay

The indirect immunofluorescence assay (IFA) was performed to detect the generation of the recombinant baculoviruses and the expression of the recombinant protein in the transfected cells. Briefly, the cells were fixed with anhydrous methanol, washed thrice with PBS, and blocked with 5% skimmed milk for 1 h at room temperature (RT). Then, the cells were washed thrice with PBST and incubated for 1 h with the Anti-His Tag Mouse Monoclonal Antibody (Abbkine, Wuhan, China, ABT2050) diluted at 1:2000. The cells were then washed thrice with PBST and incubated for 1 h with Fluorescein Isothiocyanate (FITC)-conjugated Rabbit Anti-mouse IgG Antibody (Sigma St. Louis, MO, USA, AP106F) diluted at 1:500. Finally, the specific fluorescence was observed under an inverted fluorescence microscope (Nikon, Ti-S, Minato, Japan).

### 2.4. Recombinant Protein Expression and Purification

Recombinant proteins were prepared by infecting the Sf9 cells with the recombinant baculoviruses at a multiplicity of infection (MOI) of 2.0. Seventy-two hours after infection, the cells were collected by centrifugation and then subjected to ultrasonic fragmentation. Next, the mixture was centrifuged at 2000× *g* for 10 min for separation into sediment and supernatant. A 6 × His-Tagged Protein Purification Kit (CWBIO, Taizhou, China) was used to purify the recombinant proteins in the supernatant. 

### 2.5. SDS-PAGE and Western Blot

The protein samples were dissolved in 5 × SDS-PAGE loading buffer and denatured by boiling at 100 °C. Subsequently, the samples were separated via electrophoresis and the gel was stained with Coomassie Blue. For the Western blot, the protein bands were transferred to the nitrocellulose membrane and the membranes were blocked by incubating with 5% skimmed milk for 1 h at RT, washed thrice with PBS, and incubated overnight at 4 °C with the Anti His-Tag Mouse Monoclonal Antibody (CWBIO, Taizhou, China, CW0286) diluted at 1:7000. Next, the membranes were washed thrice with PBST and incubated for 1 h at RT with the Goat anti-Mouse Secondary Antibody (Gene Company Limited, Hong Kong, China, 926-32210) diluted at 1:10,000. Finally, the membranes were washed thrice with PBST and visualized using the Odyssey Infrared Imaging System (LI-COR Biosciences, Lincoln, NE, USA).

### 2.6. Hemagglutination Activity and Hemagglutination Inhibition (HI) Assay

For the hemagglutination activity assay, the samples were serially two-fold diluted with PBS and then incubated with a 1% chicken red blood cell (RBC) suspension for 30 min at RT. The hemagglutination titer was recorded as the reciprocal of the highest dilution that caused full hemagglutination.

For the HI assay, the serum samples were preprocessed with the receptor-destroying enzyme (RDE, Seiken, Japan) for 16 h at 37 °C. This was followed by inactivation of the RDE at 56 °C for 30 min and serial two-fold dilution with PBS. Next, the diluted samples were incubated with the 4 hemagglutination units of the inactivated virus antigens for 1 h at RT. After this, the mixtures were incubated with 1% chicken RBC suspension for 30 min at RT. The HI titer was described as the reciprocal of the highest dilution that caused complete hemagglutination inhibition.

### 2.7. Thermal Stability Test

To evaluate the thermal stability of the recombinant HA proteins, we carried out a thermal stability test as described in previous research [25]. Briefly, protein samples with equal hemagglutination titers were heated at the designated temperature for 30 min, with 8 samples being processed at each temperature. After the protein samples cooled down to RT, the changes in hemagglutination titers were measured and recorded. The calculation formula for thermal stability was defined as (the remaining hemagglutination titer/the initial hemagglutination titer) × 100%.

### 2.8. Chicken Immunization and Challenge Experiment

Two-week-old SPF chickens were randomized. Two groups of chickens (N = 13 per group) were immunized subcutaneously with 60 μg of HAm and HA-WT combined with ISA 71VG adjuvant (Seppic, Paris, France). The control group received an equal volume of PBS. Blood samples were collected 14 days and 19 days post-immunization (dpi) to isolate the sera. At 21 dpi, all chickens were intranasally challenged with 10^6^ 50% egg infectious dose (EID_50_) in 200 μL of the HPAIV H7N9-E157. After the viral challenge, chickens were monitored for clinical symptoms and survival status for 14 days. At 5 days post-challenge (dpc), oropharyngeal and cloaca swabs were collected from the chickens for virus shedding detection using the SPF chicken embryo re-isolation method. Briefly, the swabs were resuspended in 1 mL of PBS supplemented with 10,000 U/mL streptomycin and 10,000 U/mL penicillin and then centrifuged at 4000× *g* for 10 min. The supernatants of each sample were used to inoculate three 9-day-old SPF chicken embryos repeatedly (100 μL/egg). After incubation for 48 h at 37 °C, the allantoic fluids were collected and measured using the hemagglutination activity assay. The swab was determined to be a positive swab if the hemagglutination titer of the inoculated chicken embryo’s allantoic fluid was higher than 4log_2_, based on references from research conducted by the Animal Infectious Diseases Laboratory of the Veterinary College of Yangzhou University [31].

The animal experiments were examined and permitted by the Animal Care and Use Committee of SCAU and performed following the guidelines.

### 2.9. Preparation and Stimulation of Chicken Splenocytes and PBMCs

At 19 dpi, chicken splenocytes and peripheral blood mononuclear cells (PBMCs) were isolated using the mononuclear cell separation kit (Tbdscience, Tianjin, China) according to the manufacturer’s protocol. The harvested cells were suspended in complete Roswell Park Memorial Institute (RPMI) 1640 medium supplemented with 10% FBS and 1% Penicillin-Streptomycin Solution (Gibco, Carlsbad, CA, USA) and then cultured in 6-well cell plates for 1 h. Subsequently, the cells were stimulated with 15 μg of the specific HA proteins or inactivated H7N9-E157 virus antigens and incubated for 12 h at 37 °C. Finally, the cells were gathered for RNA extraction and cytokine secretion detection.

### 2.10. RNA Extraction and Quantitative Real-Time PCR (qRT-PCR) Analysis

The total mRNA of the stimulated cells was extracted using a Total RNA rapid extraction kit (Feijie, Shanghai, China) and reverse transcribed into cDNA using HiScript^®^ III RT SuperMix for qPCR (+gDNA wiper, Vazyme, Nanjing, China) according to the manufacturer’s protocols. The qRT-PCR was performed to detect cytokine mRNA levels using a Bio-Rad CFX96 Deep Well Real-time system. The primer sequences of relevant genes are shown in Table 2. The change in the cytokine mRNA level was calculated using the 2^−ΔΔCt^ approach and reported as the fold difference.

### 2.11. Lymphocyte Proliferation Assay (LPA)

The cells were seeded in 96-well cell plates with complete RPMI-1640 medium, stimulated with or without 0.15 μg of the specific HA proteins or inactivated H7N9-E157 virus antigens and incubated at 37 °C for 24 h. Subsequently, an MTT Cell Viability Assay Kit (RIBOBIO, Guangzhou, China) was used to examine lymphocyte proliferation according to the manufacturer’s instructions. The results of the cell proliferation assay were reported as the stimulation index (SI), which was calculated using the following equation: SI = (OD570 of the stimulated cells − OD570 of the blank control)/(OD570 of the unstimulated cell − OD570 of the blank control).

### 2.12. Statistical Analysis

The data were reported as mean and standard deviations. Statistically significant differences between groups were evaluated using one-way ANOVA and Tukey’s multiple-comparison test and indicated as * (*p* < 0.05), ** (*p* < 0.01), *** (*p* < 0.001), or **** (*p* < 0.0001).

## 3. Results

### 3.1. Recombinant Baculoviruses Generation

**Figure 1:**
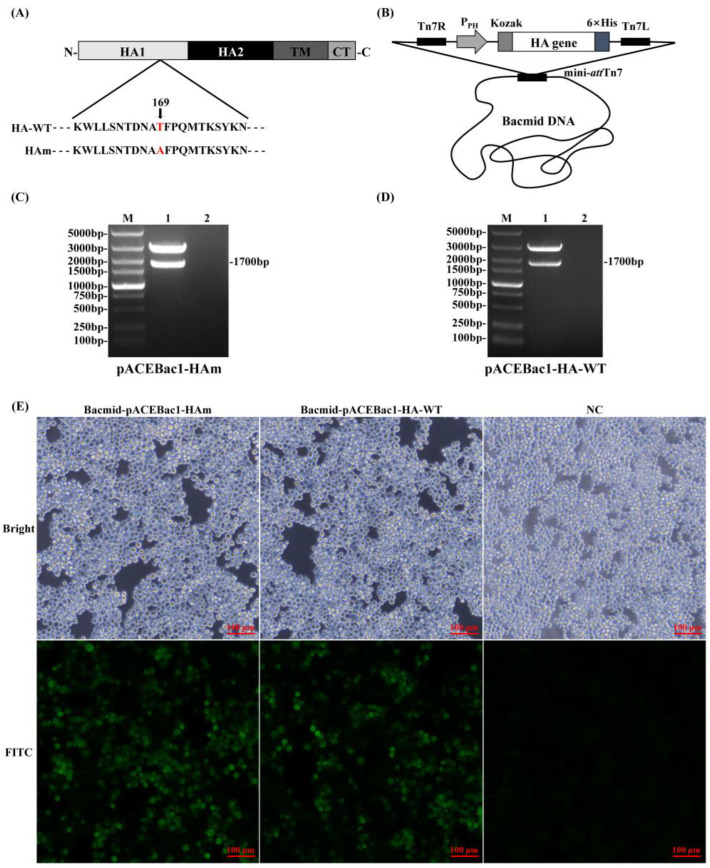
Generation and characterization of recombinant baculoviruses. (**A**) Amino acid sequences of HA-WT and Ham; the mutation site is marked as red. (**B**) A schematic diagram of HA gene modification and plasmid construction. The recombinant transfer plasmid carrying the genes of interest was transposed into the bacmid DNA. (**C**) pACEBac1-Ham restriction identification. M: DL 5000 DNA Marker; 1: pACEBac1-HAm; 2: negative control. (**D**) pACEBac1-HA-WT restriction identification. M: DL 5000 DNA Marker; 1: pACEBac1-HA; 2: negative control. (**E**) Indirect immunofluorescence assay for recombinant proteins expressed in transfected Sf9 cells.

The genes of interest were cloned into the transfer plasmid, pACEBac1, and transposed into the Bacmid DNA for transfection of the Sf9 cells (Figure 1A,B). The positive recombinant plasmids were identified by the restriction of endonuclease digestion, and the results showed that the recombinant transfer plasmids were successfully constructed (Figure 1C,D). Seventy-two hours after transfection, the transfected Sf9 cells showed increased diameters and the specific immunofluorescence was detected simultaneously, which suggested that recombinant baculoviruses were generated and could express the recombinant protein in Sf9 cells (Figure 1E).

### 3.2. Production and Characterization of Recombinant HA Proteins

**Figure 2:**
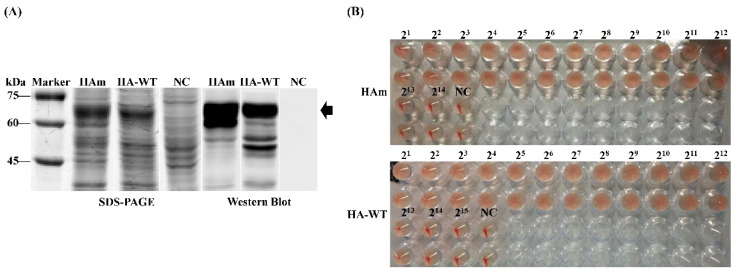
Generation and characterization of recombinant HA proteins. (**A**) SDS-PAGE and Western blot identification of recombinant HA protein expression. (**B**) Hemagglutination activity assay of the recombinant HA proteins.

The production of recombinant proteins was confirmed using SDS-PAGE and Western blot. As shown in Figure 2A, the specific protein bands of approximately 63 kDa, which conformed to the expected size of the target proteins, were detected in the centrifuged supernatant of the ultrasonicated product. For further identification, the hemagglutination activity of two recombinant proteins, in equal quantities, was detected using the hemagglutination activity assay; the results showed that the hemagglutination titers of the two recombinant proteins were both 12log_2_ (Figure 2B).

### 3.3. Analysis of the Thermal Stability of Recombinant Proteins

**Figure 3:**
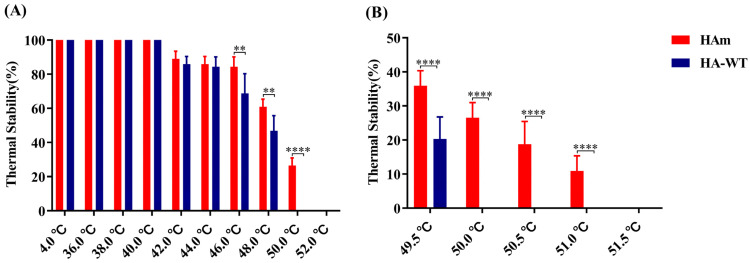
Analysis of the thermostability of recombinant HA proteins. (**A**) The protein samples were incubated at temperatures of 36–52 °C for 30 min and then the HA titers were measured after the samples cooled down to RT. (**B**) The protein samples were incubated at temperatures of 49.5–51.5 °C for 30 min, and then the HA titers were measured after the samples cooled down to RT. Error bars represent the mean ± S.D. Statistically significant differences between the two groups are indicated as ** (*p* < 0.01), or **** (*p* < 0.0001).

The HAm and HA-WT samples were simultaneously heated for 30 min at temperatures ranging from 36 to 52 °C; the hemagglutination titers were then measured. The results showed that at lower temperatures (36 to 40 °C), the hemagglutination activity of the proteins did not change (Figure 3A). However, as the treatment temperature increased, the hemagglutination activity of the proteins decreased and was not detectable when the temperature reached 52 °C. Interestingly, when the temperatures reached over 46 °C, the thermal stability of HAm was significantly higher than that of HA-WT. Specifically, at 46 °C, HAm retained nearly 85% of its hemagglutination titer, while HA-WT retained less than 70% of its hemagglutination titer; there was a significant difference between the two groups (*p* < 0.01). At 48 °C, HAm retained 60% of its hemagglutination titer, while HA-WT lost more than 50% of its hemagglutination titer; the difference between the two groups was significant (*p* < 0.01). At 50 °C, HAm retained 26.5% of its hemagglutination titer, while HA-WT lost all of its hemagglutination titers; the difference between the two groups was very significant (*p* < 0.0001).

To further verify their thermostability divergence, the temperature range was narrowed down to 49.5–51.5 °C. As expected, the data in Figure 3B revealed the divergence between them more intuitively. When the temperature increased to 49.5 °C, HAm retained 36% of its hemagglutination titer, while HA-WT retained only 20% of its hemagglutination titer. There was a very significant difference between the two groups (*p* < 0.0001). Interestingly, when the temperatures reached 50.5 °C and 51 °C, HAm still retained nearly 19% and 11% of its hemagglutination activity, respectively. In short, the results above demonstrate that HAm obtained enhanced thermostability.

### 3.4. Vaccine Immunization-Induced Cross-Reactive HI Antibody Response

**Figure 4:**
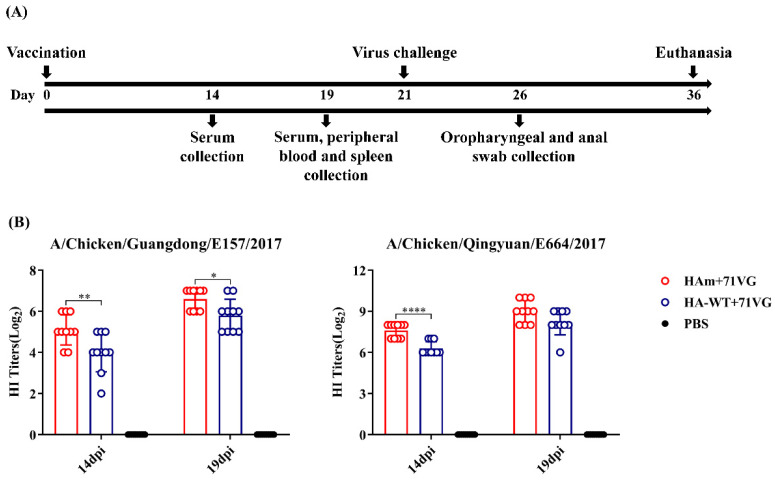
Animal experimental process and HI antibody levels. (**A**) Schematic diagram of the animal experiment. (**B**) HI antibody levels of vaccinated chickens were identified at 14 days and 19 days post-immunization (dpi) using a HI assay. Error bars represent the mean ± S.D. Statistically significant differences between groups are indicated as * (*p* < 0.05), ** (*p* < 0.01), or **** (*p* < 0.0001).

To verify whether the enhancement in thermal stability improves the immunogenicity of HAm, chickens were subcutaneously immunized once with two proteins combined with ISA 71VG (Figure 4A). The data in Figure 4B show that at 14 dpi, vaccines induced an effective HI antibody response against H7N9-E157, and the HAm group induced a significantly higher HI titer than the HA-WT group (*p* < 0.01). The mean HI titer of the HAm group was also higher than that of the HA-WT group against H7N9-E664, with a very significant difference between the two groups (*p* < 0.0001).

At 19 dpi, both groups induced higher HI antibody responses. The HAm group had significantly higher HI titers against H7N9-E157 than the HA-WT group (*p* < 0.05). The mean HI titers of the HAm and HA-WT groups were 9log_2_ and 8.2log_2_, respectively, against H7N9-E664, but there was no statistically significant difference between the two groups. The results of the HI assay demonstrated that a single dose of the HAm vaccine could elicit a higher cross-reactive HI antibody response against the antigenically divergent H7N9 viruses more rapidly than the HA-WT vaccine.

### 3.5. Lymphocyte Proliferation and Cytokine Secretion Levels

**Figure 5:**
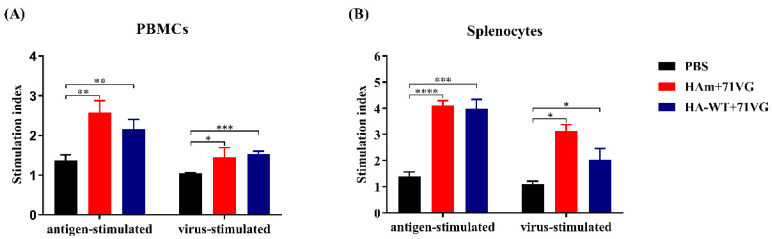
Proliferation of PBMCs and splenocytes. (**A**) Stimulation index of the PBMCs. (**B**) Stimulation index of the splenocytes. PBMCs and splenocytes were prepared from the chickens at 19 days post-immunization (dpi). The stimulation index of mononuclear cells in response to the HA protein was analyzed using the MTT Cell Viability Assay Kit. Error bars represent the mean ± S.D. Statistically significant differences between groups are indicated as * (*p* < 0.05), ** (*p* < 0.01), *** (*p* < 0.001), or **** (*p* < 0.0001).

As shown in Figure 5, both vaccine groups induced significantly greater proliferation of PBMCs and splenocytes after specific stimulation compared with the PBS group. The HAm group induced a level of lymphocyte activation similar to the HA-WT group, and there was no statistically significant difference between them.

### 3.6. Cytokine Secretion Levels

**Figure 6:**
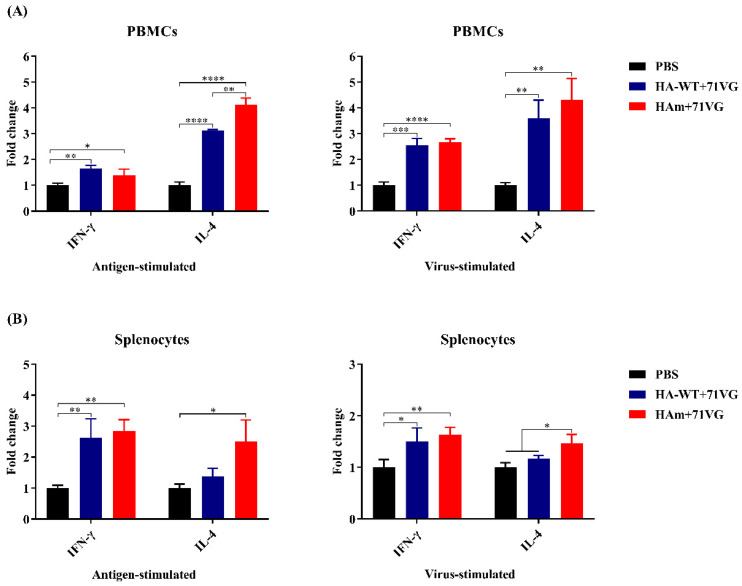
Cytokine expression levels in the PBMCs and splenocytes of chickens. (**A**) mRNA levels of IFN-γ and IL-4 in the PBMCs of chickens. (**B**) mRNA levels of IFN-γ and IL-4 in the splenocytes of chickens. PBMCs and splenocytes were isolated from chickens 19 days post-immunization (dpi) and mRNA levels of IFN-γ and IL-4 in the PBMCs and splenocytes post-stimulation were determined using qRT-PCR. Error bars represent the mean ± S.D. Statistically significant differences between groups are indicated as * (*p* < 0.05), ** (*p* < 0.01), *** (*p* < 0.001), or **** (*p* < 0.0001).

Levels of the cytokines IFN-γ and IL-4, associated with Th1-type and Th2-type immune responses, respectively, were analyzed to determine the effect of recombinant protein vaccines on stimulating cellular immune response [32,33]. The data in Figure 6A show that after antigen and virus stimulation, an effective secretion of IFN-γ and IL-4 was induced from PBMCs in the vaccine groups. Notably, after antigen stimulation, the levels of IL-4 from PBMCs in the HAm group were significantly higher than those from PBMCs in the HA-WT group (*p* < 0.01). Similarly, for the cytokine secretion levels of the splenocytes (Figure 6B), the results showed that after antigen and virus stimulation, an effective secretion of IFN-γ and IL-4 was provoked and after virus stimulation, the IL-4 level of the HAm group was significantly higher than that of the HA-WT group (*p* < 0.05).

### 3.7. Protective Efficacy of Recombinant HA Protein Vaccines in Chickens

**Figure 7:**
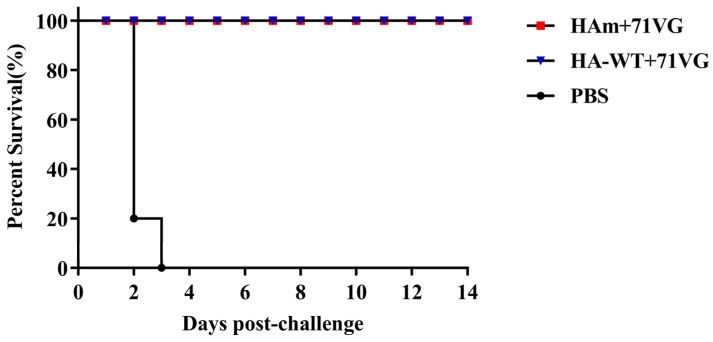
Percent survival of chickens. All chickens in the PBS group died within three days of being challenged with a heterologous H7N9 AIV A/Chicken/Guangdong/E157/2017 at a dose of 10^6^ EID_50_, while 100% of the chickens in the HAm group and the HA-WT group survived over the whole 14-day observation period.

At 21 dpi, a viral challenge with heterologous AIV H7N9-E157 was performed to investigate the protective efficacy of the vaccines in chickens. After the viral challenge, the clinical signs of infection in chickens were monitored daily and virus shedding was examined 5 dpc (Figure 4A). The results showed that both vaccine groups had 100% clinical protection (Figure 7). Virus shedding results showed that at 5 dpc, 20% (2/10) of the chickens in the HA-WT group shed the virus through the oropharynx or cloaca, and only 10% (1/10) of the chickens in the HAm group shed the virus through the oropharynx (Table 3). By contrast, chickens in the PBS group began to show evident onset symptoms from 2 dpc, including breathlessness and nasal-ocular discharge. 80% of the chickens in the control group died of the viral challenge 2 dpc, while the remaining chickens died 3 dpc (Figure 7).

## 4. Discussion

For subunit vaccines, where antigen protein is the main component, the stability of the antigen protein is one of the most significant factors. Enhancing the stability of the antigen protein could not only slow down the degradation of the antigen protein but may also heighten the immunogenicity of the antigen protein. Research has reported that the HA of wild-type H3N2 has higher thermostability than mutant HA containing mutations in the TM, with the H1, H5, H7, and H9 HA proteins comprising the TM of H3N2 showing strengthened thermostability and correspondingly increased hetero-protection [20,21,22,23,24,25,26,27]. Another research team designed a stem-region immunogen (H1HA6) and then conducted random mutagenesis and surface display of yeast and showed that H1HA6P2, which has no heterotrimeric sequence, possesses a trimerized conformation similar to that of the native protein and markedly increased thermostability, as well as enhanced binding to several broadly neutralizing-related antibodies [34]. These previous studies have intimated an important biological link between HA stability and immunogenicity. Additionally, the optimized thermal stability may contribute to the practical application of the antigen during vaccine manufacture, as sometimes the vaccine production process involves some steps that are not conducive to maintaining protein activity. For example, the ultrasonic crushing process and the vaccine emulsification process may generate heat. The production of subunit vaccines from baculoviruses may involve operational steps that require long-term treatment at higher temperatures, such as baculovirus inactivation. The development of heat-stable vaccines is not only an urgent need in the field of human vaccines but also an issue that needs to be considered in the development of veterinary vaccines, as it can effectively avoid the waste of vaccine resources and promote the application of vaccines in remote areas [35].

In this study, one mutant HA protein was generated by substituting amino acid site 169 in the H7N9-16876 HA protein. Initially, we evaluated the impact of the mutation on thermal stability. The data showed that the HAm acquired greater thermostability compared with the HA-WT. As shown in Figure 3, the difference in stability between the two proteins gradually increased as the treatment temperature rose, especially when the temperature reached over 48 °C. Interestingly, we noticed that the T169A mutation may abrogate the potential glycosylation site N167, which may also contribute to exposing the potential epitope masked by glycans or increase the thermostability of HA protein; however, more detailed research is still needed to verify this observation. Subsequently, the HI antibody titers of the sera derived from the immunized chickens were measured to evaluate the immunogenicity of two different antigens. Compared with chickens vaccinated with HA-WT, the chickens vaccinated with the HAm showed significantly higher cross-reactive HI antibody titers against two variant viruses: H7N9-E157 and H7N9-E664 (Figure 4B). 

In addition to stimulating the humoral immune response, an ideal vaccine could also irritate the cellular immune response [36]. Appropriate cooperation with a suitable adjuvant is a feasible strategy that can effectively promote the cellular immune response. In our study, a commercial mineral oil-based W/O emulsion adjuvant, ISA 71 VG, was selected and combined with the recombinant antigens to increase the potency and effectiveness of immunization. Cytokine secretion from the splenocytes and PBMCs of the vaccinated chickens and the proliferation of these cells were evaluated. The results showed that the cells from the vaccine groups showed significantly higher proliferation activity compared with cells from the PBS group (Figure 5). For the cytokine mRNA levels, IFN-γ and IL-4 levels of splenocytes and PBMCs from the immunized chickens were significantly higher than the levels of the PBS group (Figure 6). These results suggested that the antigen-specific lymphocytes were activated after stimulation following vaccine immunization. Notably, the IL-4 levels in the HAm group were significantly higher than in the HA-WT group, consistent with serum antibody levels, which may indicate that an appropriately elevated IL-4 level in the HAm group helps promote the humoral immune response. 

Regarding the protection potency of the vaccines, our data showed that vaccines provided chickens with complete clinical protection against the lethal attack of the heterologous virus, H7N9-E157. The result of the virus shedding test demonstrated that both vaccines effectively inhibited virus shedding, with only 10% (1/10) and 20% (2/10) of the chickens shedding residual viruses via the oropharynx or cloaca, respectively. However, there was no significant difference in protective effects between HAm and HA-WT. One reason may be that the number of animals in this study was relatively small; another reason may be that the immune doses used were relatively high. More importantly, we still need to improve the potency and breadth of the protection of our vaccines and decrease viral shedding. The ideal vaccine should not only protect animals from death when viruses attack but also adequately reduce virus shedding to effectively inhibit the spread of the virus and provide extensive and comprehensive protection. In the future, we could further optimize the antigen design through other strategies such as introducing it to the surface of self-assembled ferritin nanoparticles. Using this novel strategy, researchers found that the fusion of ferritin and the ectodomain of HA protein could self-assemble nanoparticles with improved potency and breadth of immune protection [37,38,39]. Similarly, recent studies on the development of SARS-CoV-2 vaccines showed that fusing spike-functionalized or receptor-binding domain (RBD) to ferritin could generate nanoparticles, and the nanoparticle vaccine could elicit robust humoral and cellular immune responses in mice and rhesus monkeys [40]. Promoting the trimerization of HA protein [41] and forming biomimetic VLPs [42] are also promising vaccine design strategies. More importantly, in order to effectively inhibit virus excretion and transmission, multiple strategies will need to be applied jointly. Among them, exploring other vaccine delivery routes is one of the key strategies. At present, the approved avian influenza vaccine is administered via intramuscular injection, which cannot effectively induce mucosal immunity, especially in the respiratory tract. However, influenza virus infection usually occurs in the upper respiratory tract at first, and this limitation may reduce the effectiveness of these vaccines against anti-infection and inhibition of virus transmission. In fact, the mucosal IgA response is elicited earlier than the systemic IgG response and plays a role in the early stage of infection, inhibiting the entry of the virus into epithelial cells and preventing the assembly and release of virus particles [43]. Therefore, intranasal delivery of the vaccine to induce respiratory IgA response may be a feasible reference strategy [44,45].

The comprehensive results of our research indicate that the mutant HA protein vaccine may be a safe and effective avian influenza subunit vaccine candidate. The 2-week-old chickens can also acquire good immune protection after being immunized with the vaccines. Interestingly, we found that mutated antigen proteins have higher thermal stability than wild-type antigens, which may be a potential advantage. However, it must be acknowledged that the amino acid modification strategy may have limitations as it may not always improve stability and may also reduce the immunogenicity of antigens. Therefore, when modifying amino acids, analysis of the protein structure and the epitope is also significant. Although there remains a lot of work to be done, we hope that our study can provide some reference for related research.

## 5. Conclusions

In summary, our research successfully generated a mutant HA protein derived from the AIV H7N9-16876 using overlapping PCR and BEVS. The mutant possesses good hemagglutination activity, with a hemagglutination titer of 12log_2_. Compared with the wild-type HA protein, the mutant had significantly enhanced thermostability, which may facilitate its practical application and be an advantage in protein vaccine production. A higher cross-reactive HI antibody response was elicited after immunizing chickens with a single dose of the mutant protein vaccine. Additionally, stronger lymphocyte proliferation and secretion of IFN-γ and IL-4 were also detected. Importantly, both recombinant HA protein vaccines provided 100% clinical protection against lethal challenges with heterologous AIV H7N9-E157 and provided an effective rate of virus-shedding inhibition (80% and 90%, respectively).

## Figures and Tables

**Table 1:** Primer sequences for overlapping PCR.

Primer	Sequence
F1	5′-CGGGATCCGCCACCATGAACACTCAGATCC-3′
R1	5′-AGCGTTGTCGGTGTTGGACAG-3′
F2	5′-TGTCCAACACCGACAACGCTG-3′
R2	5′-CGGAATTCTTAGTGATGGTGGTGGTGG-3′

**Table 2:** Primer sequence for quantitative real-time PCR.

Gene	Primer Sequence	Product Size	Accession No.
β-actin	F: 5′-CGTTGTTGACAATGGCTCCG-3′	122 bp	NM_205518.2
	R: 5′-GGCCCATACCAACCATCACA-3′		
IFN-γ	F: 5′-AGCTCCCGATGAACGACTTG-3′	118 bp	NM_205149.2
	R: 5′-CTCCTCTGAGACTGGCTCCT-3′		
IL-4	F: 5′-TGCTTACAGCTCTCAGTGCC-3′	215 bp	NM_001007079.2
	R: 5′-AGCTGACGCATGTTGAGGAA-3′		

**Table 3:** Virus shedding by the chickens 5 dpc.

Group	Oropharyngeal Swab (Virus Shedding Number/Total Number)	Cloacal Swab (Virus Shedding Number/Total Number)	Total Virus Shedding Number/Total Number
HAm + 71VG	1/10	0/10	1/10
HA-WT + 71VG	1/10	1/10	2/10
PBS	-	-	-

## Data Availability

All datasets generated from this study are included in the article.
